# Screening antibiofilm activity of invasive plants growing at the Slope Merapi Mountain, Central Java, against *Candida albicans*

**DOI:** 10.1186/s12906-023-04044-2

**Published:** 2023-07-12

**Authors:** Sufi Desrini, Marion Girardot, Christine Imbert, Mustofa Mustofa, Titik Nuryastuti

**Affiliations:** 1 Department of Pharmacology, Faculty of Medicine, Universitas Islam Indonesia, Yogyakarta, Indonesia; 2 Doctoral Program of Faculty Medicine, Public Health, and Nursing, Universitas Gadjah Mada, Yogyakarta, Indonesia; 3 Laboratoire Ecologie Et Biologie Des Interactions - UMR CNRS 7267, Université de Poitiers, Poitiers, France; 4 Department of Pharmacology and Therapy, Faculty of Medicine, Public Health, and Nursing, Universitas Gadjah Mada, Yogyakarta, Indonesia; 5 Indonesia Biofilm Research Collaboration Center UGM-BRIN, Yogyakarta, Indonesia; 6 Department of Microbiology, Faculty of Medicine, Public Health, and Nursing, Universitas Gadjah Mada, Yogyakarta, Indonesia

**Keywords:** Invasive plants, *Candida albicans*, Antifungal, Antibiofilm, *Mimosa pudica*

## Abstract

**Background:**

*Candida albicans* causes high-mortality candidiasis. Antifungal drug resistance demands the development of virulence factor-targeting drugs, particularly antibiofilm. This study screened the effects of five invasive plants growing in Indonesia (*Mimosa pudica*, *Lantana camara*, *Acacia mangium*, *Ageratina riparia*, and *Mikania micrantha*) against *C. albicans* biofilms. Antifungal activity, antiphospholipase activity, biofilm morphology of *C. albicans,* and cytotoxic capacity were also evaluated.

**Methods:**

Maceration was used to extract the plants, and the most active extract inhibiting the biofilms was fractionated using liquid–liquid fractionation. Antibiofilm activity was determined by a colorimetric assay, MTT. Antifungal activity was tested using the broth microdilution method. A phospholipase assay was performed using the egg-yolk agar method. Influence on the *C. albicans* morphology was assessed using scanning electron microscopy (SEM). The cytotoxic effect was carried out against Vero and HeLa cell lines.

**Results:**

*M. pudica* extracts showed the most potent antifungal efficacy with minimum inhibitory concentration (MIC) of 15.62 µg/mL and 7.81 µg/mL for aerial parts and roots, respectively. At high concentrations (500 µg/mL and 250 µg/mL), ethanol extract of *M. pudica* aerial parts strongly inhibited the phospholipase activity. Ethyl-acetate fraction of *M. pudica* aerial parts demonstrated the most potent antibiofilm activity against 24 h old biofilm of *C. albicans* with an inhibitory concentration (53.89%) of 62.5 µg/mL showed no cytotoxicity in both Vero and HeLa cells. This fraction affected the morphology of *C. albicans* and contained promising compounds for inhibiting the 24 h old biofilm of *C. albicans*.

**Conclusions:**

Invasive *M. pudica* plant inhibited the growth of planktonic *C. albicans* cells and its ethyl acetate fraction decreased the metabolic activity of *C. albicans* biofilms. This result demonstrates the potential of invasive *M. pudica* plant to reduce biofilm-associated candida infection.

**Supplementary Information:**

The online version contains supplementary material available at 10.1186/s12906-023-04044-2.

## Background

*Candida albicans* is the yeast that usually can be detected in healthy humans without causing health issues. However, when the immune system is compromised (e.g., long-term exposure to antibiotics, utilization of indwelling medical devices, and post-surgery), this yeast can penetrate the natural host barriers, invade the bloodstream, and intensely attack various organs, leading to invasive candidiasis (bloodstream infection/candidemia) and deep-seated infection with or without candidemia) that seriously threaten life [1, 2]. Indeed, the global burden of invasive candidiasis remains high, with candidemia-causing *C. albicans* being the most prevalent, either in the general population or in hospitals [3]. In Indonesia, approximately 7.7 million people have a serious fungal infection each year with the assumption of the candidemia incidence (the common invasive infection) was 10/100,000 [4]. In Europe, the number of candidemia between January 2000 and February 2019 was approximately 79 cases per day, with the fatal cases being around 29 patients on day thirty [5]. According to the Centers for Disease Control and Prevention’s (CDC) surveillance data, the mortality of candidemia in hospitals is around 25% [6].

The pathogenicity of *C. albicans* depends on some virulence factors such as biofilm formation, and secretion of extracellular enzymes [2]. Concerning biofilm formation, unicellular *C. albicans* cells must adhere to indwelling medical devices before infection, for then accumulate with other cells to form basal layers. Following initial adherence, most of the adherent yeast cells switch to the hyphal form, secrete extracellular polymeric substances, and get encapsulated in a layer of hydrogel, namely extracellular matrix, forming a physical barrier between the community and the extracellular environment. This process continues to thick and grows into a mature biofilm with a three-dimensional structure [7, 8]. Regarding phospholipase (one of the extracellular hydrolytic enzymes), it facilitates the adherence and invasion of *C. albicans* cells to the host epithelium by hydrolyzing phospholipids and peptide bonds, which play and regulate an essential role in multiple physiological processes on the human body such as immune system and stress tolerance [9–12].

The presence of virulence factors, especially biofilm formation, is associated with *C. albicans* resistant to the majority of antifungal drugs. Although biofilm resistance is multifactorial and mechanistically complex, the role of the extracellular matrix as a physical barrier may account for the high levels of resistance displayed by *C. albicans* biofilms [13, 14]. Al-Fattani and Douglas (2006) identified a correlation between matrix abundance and levels of fluconazole and amphotericin B resistance [15]. Moreover, the newest class of antifungal drugs, echinocandin, revealed the reduction susceptibility (resistance) against clinical and laboratory strains of *Candida albicans* [16, 17]. In the context of phospholipase enzyme, Ying and Chunyang, 2011 reported that there was a correlation between high phospholipase activity and resistance to antifungal drugs by increasing the expression of phospholipase B1 mRNA and protein [18]. Another study showed that some antifungal agents such as nystatin, fluconazole, and micafungin had a low reduction (approximately under 5%) of phospholipase activity [19].

Taken together, the high morbidity and mortality of invasive candidiasis and the great capability of *C. albicans* to resist antifungal agents demand the discovery of new drugs to protect humans against *Candida* infections, especially those associated with a biofilm. By incorporating traditional knowledge of plants as remedies into the drug discovery process, natural products can serve as a source of new drugs or active pharmaceutical ingredients. Indeed, the use of plants as medicine has a lengthy history, and remarkably, many drugs have already been derived from plants. However, global demand for medicinal plants has endangered native plants, contributing to biodiversity loss and depletion of natural resources critical to human health [20]. Moreover, the situation is worsened by the presence of invasive plants which entered and established in the new environment from outside of their natural habitat and caused environmental, economic, and/or human harm [21]. One of the most serious threats posed by invasive plants to the environment is the disruption of entire ecosystems. According to the United Nations (UN) Intergovernmental Platform for Biodiversity and Ecosystem Services (IPBES), the impacts of invasive plants are often severe for native species and especially for endemic species. Native species are estimated to have lost at least 20% of their original abundance, and even more in hotspots of endemic species [22]. Meanwhile, Indonesia, one of the world's richest nations in terms of biodiversity, with around 30,000 plant species and 9,600 medicinal plants [23], is known for the high rates of loss of diversity in the world that are caused by the introduction and spread of invasive plants in various Indonesian ecosystems [24]. Nevertheless, despite the negative effects caused by invasive plants, there are positive aspects, especially in the health sector. Numerous studies have documented the use of invasive plants in traditional medicine. For example, *Mimosa pudica* leaves (native of tropical America) are used to treat toothache and low libido in men, respectively, on Rodrigues Island of the Indian Ocean and Kurukshetra District, India [25, 26]. The leaves of *Lantana camara* (native to tropical America) have been reported to treat many diseases such as tuberculosis in South-Western Uganda, ulcers, swelling, and microbial infections in India [27–29]. Furthermore, Máximo et al., 2020 demonstrated the pharmaceutical potential of invasive plants that have produced compounds. They described the potential of invasive plants such as *Carpobrotus edulis*, *Hakea salicifolia*, *Hakea sericea*, *Oxalis pes-caprae,* *Phytolacca americana,* and *Ageratina adenophora* as sources of bioactive metabolites ranging from antioxidant, antimicrobial, and anticholinesterase to neuroprotective and antiproliferative [30]. Taking those matters into account, the authors put those main ideas of drug development into practice by utilizing five invasive plants growing in Indonesia and screening their antibiofilm activity against *C. albicans*. In addition, this study also screened for antifungal and antiphospholipase activity. Notably, these five plants are listed by the Ministry of Environment and Forestry as invasive plants which might become big threats to agriculture, forests, and other resources in Indonesia [31]. To the best of our knowledge, this study is the first to evaluate the efficacy of Indonesian invasive plants in inhibiting *C. albicans* virulence factors.

## Materials and methods

### Plant materials

*M. pudica* aerial parts (= *M. pudica*'s structures above ground, including leaves, flowers, and stem), *M. pudica* roots, *L. camara* leaves,* A. mangium* leaves, *A. riparia* leaves, and *M. micrantha* leaves were used in this study and collected from the slopes of Merapi mountain, Indonesia (GPS positioning: between -7.5719346390002675, 110.43219680357387 and 7.5719346390002675, 110.43219680357387). The identification of plants was conducted by Dr. Djoko Santosa (Department of Biological Pharmacy, Faculty of Pharmacy, Universitas Gadjah Mada) and by Anggityas Puspita Suci, S. Farm, Apt. (Merapi Farma Herbal) (identification number: 13.17.09). The voucher specimens of *A. riparia*, *M. micrantha*,* A. mangium*,* M. pudica*, and *L. camara* were 43AR-1, 43MM-2, 43AM-4, 43MP-5, and 43LC-6, respectively, and were deposited at the Department of Biology Pharmacy, Faculty of Pharmacy, Universitas Gadjah Mada.

### Extraction of plant materials

The plants were shade-dried for seven days and powdered using a grinder. Afterward, the powder of each plant was processed for the preparation of ethanol (Merck, Darmstadt, Germany) or methanol extract (Merck, Darmstadt, Germany) by the maceration process as described previously with modification [32]. For this purpose, 100 g of plant powder were macerated in 500 mL of ethanol or methanol for three days with regular shaking. After filtration using the Buchner funnel, the residues were re-macerated using fresh solvents for three days. All filtrates obtained with the same solvent were pooled, filtered through filter papers (Whatman filter paper no. 1) in the Buchner funnel, and dried using a rotary evaporator (Heidolph, Schwabach, Germany). Samples were stored in a refrigerator at -20 °C until further experiments.

### Liquid–liquid fractionation (LLF)

The extract with the highly active extract was subjected to liquid–liquid fractionation (LLF) according to the method as described previously with modifications [33]. For this purpose, the organic solvents (analytical grade, Merck, Darmstadt, Germany), in order of increasing polarity, were n-hexane, chloroform, ethyl-acetate, n-butanol, and double-distilled water (ddH20). Before partition, 5 g of extracts were solubilized in 10 mL of ethanol (Merck, Darmstadt, Germany) and 90 mL of ddH_2_0. The solubilized extract was then partitioned with 100 mL of n-hexane, shaken, and then the *n*-hexane layer was separated. This process was carried out three times. Chloroform, ethyl-acetate, and n-butanol were processed following the same method. Each partition was performed three times, and the same eluents were pooled and dried using a rotary evaporator. Each obtained fraction was recorded as the total yield.

### Phytochemical profiling

#### Liquid Chromatography-Mass Spectrometry (LC–MS)

The chemical composition of the best active extract was qualitatively screened and analyzed by Liquid Chromatography-Electrospray Ionization-Mass Spectrometry (LC–MS-ESI–MS) using Acquity UPLC I-Class coupled with XEVO G2-XS QTOF (Waters, MA, USA) mass spectrometer. The column was ACQUITY UPLC® BEH C8, 1.7 µm, 2.1 × 100 mm. The mobile phase was composed of solvent A: water with 0.1% formic acid, and solvent B: acetonitrile containing 0.1% formic acid. The flow rate was set at 0.3 mL/min with a 1 μL injection volume. The parameters of MS/MS were optimized as follows: ionization type: ESI; Start Mass: 50.00 m/z; End Mass: 1200.00 m/z; Polarity: Positive. The screening process for constituents was performed with the UNIFI software, which contains a mass spectrum library of natural chemical constituents from the waters database [34].

#### High-Resolution Mass Spectrometer (HRMS)

Thermo ScientificTM DionexTM Ultimate 3000 RSLCnano UHPLC (ultra-high-performance liquid chromatography) and Q ExactiveTM High-Resolution Mass Spectrometer (ThermoFisher, MA, USA) were used to screen and discover non-targeted chemical compounds from the fraction showing the best antibiofilm activity. The mobile phase was composed of solvent A (water with 0.1% formic acid) and solvent B (acetonitrile with 0.1% formic acid). The programming for the gradient mode was as follows: at t = 0–15 min, B 5%; at t = 16–20 min, B 90%; at t = 21–25 min, B 90%. The analytical column used was Phenyl-Hexyl 100 × 2.1 mm with a flow of 0.20 mL/min and an injection volume of 5 µl. MS1 was rendered at 70,000 FWHM, whereas MS2 was rendered at 17,500 FWHM. This experiment utilized Heated Electrospray Ionization (H-ESI) in both positive and negative modes. The spray voltage used was 3.8 kV. The flow rates for Sheath gas and Aux gas were 15 and 7, respectively. The capillary temperature was 250 °C. The mass range used was between 50 and 750 m/z. Thermo Scientific ™ Compound Discoverer Software was used for identifying the compounds.

### Strains and growth conditions

*C. albicans* ATCC 10231 was used as a reference strain, and two isolates, CI-SPTM and CI-CVX which were recovered, respectively, from sputum isolated from pulmonary disease and from cervical swab specimens of vulvovaginal candidiasis patients at two different hospitals in Yogyakarta, Indonesia, were kindly provided by the Microbiology Department, Faculty of Medicine at Universitas Gadjah Mada, Indonesia. *C. albicans* were grown in Sabouraud dextrose agar (SDA) (Himedia, Maharashtra, India) at 37 °C for 24 h.

### Antifungal susceptibility test

As per the M27-A3 protocol of the Clinical and Laboratory Standards Institute (CLSI), the broth microdilution method was used to evaluate the minimum inhibitory concentration (MIC) of the samples against *C. albicans* [35]. Each reference or clinical isolate of *C. albicans* was streaked on SDA plate. The plates were incubated for 24 h at 37 °C. Then several colonies from these cultures were picked up, and five colonies of around 1 mm diameter were suspended in sterile saline solution (0.85% NaCl) and adjusted to 0.5 McFarland standard (equivalent to 1–5 × 10^6^ cells/mL) and then diluted at 1:50, followed by a 1:20 dilution in Roswell Park Memorial Institute medium (RPMI) (Himedia AT180, Maharashtra, India), which contained 0.165 M l^−1^ 3-(N-morpholino)propanesulfonic acid (MOPS) buffer to obtain a suspension of approximately 1-5 × 10^3^ cells/mL. The sample stocks (20 mg/mL) were prepared by weighing 20 mg of extract in a sterile Eppendorf tube and diluting it in 1 mL of 25% dimethyl sulfoxide (DMSO). The 100 µL of working yeast suspension was then added to wells of 96-well microtiter plates (Corning®, NY, USA) containing 100 µL of a serial two-fold dilution in RPMI 1640-MOPS medium of extracts. The final concentrations obtained ranged between 1000 and 3.9 µg / mL. Some wells were preserved for controls: non-treated yeasts (negative control), yeasts treated by fluconazole (positive control) (Sigma St. Louis, MO, USA), and yeasts treated by DMSO 2% (DMSO control). The test was run in triplicate and repeated at least twice. The determination of MIC was conducted according to the CLSI procedure: each well was assigned a numeric rating of 0 (visually clear), 1 (a rather foggy), 2 (significant decrease in visible growth), 3 (slight decrease in visible growth), or 4 (zero reduction in visible growth). Based on numerical scales, the lowest concentration that significantly inhibited visible growth was designated as the MIC_50_ (scale of 2) [35].

By subculturing one loopful (10 μL) of the solution from the wells without turbidity on SDA, the minimum fungicidal concentration (MFC) was ascertained. After an incubation period of 24 h, the minimal fungicidal concentration (MFC) was determined to be the lowest concentration that resulted in no growth or maximum three colonies growth (> 99.9%) on the subculture.

### Antibiofilm assay

Colorimetric assays are tools that are reasonably simple to perform, very useful for determining yeast viability, and reveal a great association between cellular density and metabolic activity, allowing for semiquantitative evaluation of biofilm formation [36]. Therefore, to evaluate the metabolic activity of mature *C. albicans* biofilm, the colorimetric assay (MTT) was carried out according to the method of Prazynska and Gospodarek, 2014 with a few modifications [37]. For this purpose, yeast was first cultured on the SDA agar plate for 48 h. Thereafter, four loopfuls of this culture were transferred to 30 mL of Yeast Extract-Peptone-Dextrose (YPD) medium (Difco, Detroit, MI, USA) and cultured at 37 °C without shaking overnight. This culture was then centrifuged at 3000 g for 10 min, rinsed twice in 0.1 M phosphate-buffered saline (PBS, pH 7.2, GIBCO, New York, United States), standardized to 0.5OD_600_ (equivalent to 3 × 10^7^ CFU/mL), and diluted to get a final concentration of 1 × 10^6^ CFU/mL. The 100 µL of yeast suspension was transferred into a sterile, untreated 96-well polystyrene plate (Costar, Corning, USA), incubated for 24 h at 37 °C. Then, after 24 h of incubation, the non-adherent yeasts were removed by washing them twice with 0.2 mL sterile PBS. Two-fold serial dilutions of the extracts/fractions (between 1000 and 3.9 µg mL^−1^) prepared in YPD (Difco™ YPD Broth, USA) medium were added to each well-containing biofilm, and the microplates were incubated at 37 °C for 24 h. Further, the wells were washed twice with 0.2 mL PBS after 24 h incubation at 37 °C. Then, the wells were filled with 100 μL of MTT solution (5 mg/mL in PBS) and left at 37 °C for 90 min. Then, the solution was taken out of the incubation chamber, and the formed formazan was dissolved in 100 µL of isopropanol-HCl solution. Solubilized formazan color was measured using a microplate reader at a wavelength of 550 nm. The inhibition percentage for each concentration of the samples was calculated according to the following formula:

$$100\;-\;\left[\left(100\;\times\;\overline{\mathrm X}\;\mathrm{absorbance}\;\mathrm{of}\;\mathrm{the}\;\mathrm{treated}\;\mathrm{cells}\right)\;/\;\overline{\mathrm X}\;\mathrm{absorbance}\;\mathrm{of}\;\mathrm{cells}\;\mathrm{control}\right]$$

This study denoted a high or poor activity for above or under 50%, respectively. Inhibition percentages were calculated based on a minimum of two independent experiments with three replicates.

### Qualitative analysis-scanning electron microscopy (SEM)

*C. albicans* ATCC 10231 biofilms (control and treated cells), as described previously in the antibiofilm assay section, were prepared on Thermanox™ polystyrene coverslips (Nunc™ Thermanox™). Briefly, after 24 h, the coverslips of control and treated biofilms were washed twice with PBS and fixed with glutaraldehyde and 0.1 N PBS for 1 h at room temperature. The coverslips were washed with PBS and dehydrated in ethanol solutions (50, 70, and 90% for 10 min). After that, coverslips were air-dried overnight in a desiccator before gold sputter coating. With a scanning electron microscope (JSM-6510LA, JEOL-USA), the morphology of *C. albicans* biofilms was examined. This procedure was modified from Pereira et al. 2016 [38].

### Phospholipase assay

A phospholipase assay was performed using the egg-yolk agar method [39]. The egg-yolk agar medium contained 13 g of SDA, 11.7 g of NaCl, 0.11 g of CaCl_2_, and 8% of sterile egg-yolk emulsion (Merck, Darmstadt, Germany) in 184 mL of distilled water. After *C. albicans* ATCC 10231 was subcultured in SDA agar for 24 h, the cells (2 × 10^6^) were cultured in YPD medium using the tested extract (treated cells) for 24 h at 37 °C. The cell control (not treated) was included in this assay. In a petri dish with a 90 mm diameter, a 5 μL suspension containing 10^6^ yeast cells (treated and control cells) was plated on the surface of an egg-yolk medium and left to dry at room temperature. Afterward, the plates were incubated for seven days at 37 °C. When a precipitation zone (production of phospholipases) was visible around the *C. albicans* colony area, the phospholipase activity (Pz index) was established*,* and the formula employed to calculate the phospholipase production was: Pz = Diameter of the colony / (Diameter of the colony + precipitation zone).

The Pz values were categorized as follows: 1 (negative activity); 0.90–0.99 (very low activity); 0.80–0.89 (low activity); 0.70–0.79 (high activity); and ≤ 0.69 (very high activity).

All experiments were performed in duplicate, twice on different days.

### Cytotoxicity assay

The fraction displaying the most potent antibiofilm activity was studied to evaluate its cytotoxicity via 3-(4,5-dimethylthiazol-2-yl)-2,5-diphenyltetrazolium bromide (MTT assay). For this purpose, the protocol of the cytotoxicity assay was modified from Nugroho et al., 2013 [40]. The Vero and HeLa cell lines were used in this assay to assess whether the fraction is more selective for antibiofilm activity or more toxic for both cells (normal and cancer cells). The Vero and HeLa cell lines were obtained from the Department of Parasitology, Faculty of Medicine, Universitas Gadjah Mada, Indonesia. The Vero and HeLa cells were grown in Dulbecco’s Modified Eagle’s Medium (Gibco, NY, USA) with 2 mM glutamine, containing 10% fetal bovine serum (FBS) (Gibco, NY, USA) and RPMI 1640 (Merck, Darmstadt, Germany) with 10% FBS (Gibco, NY, USA), respectively. Briefly, the cells were plated in 96-well microplate wells (2.0E + 04 cells per well) and incubated for 24 h (5% CO_2_; 37 °C). The two-fold serial dilutions of the studied fraction were added into each well (except for medium and control wells), with concentrations ranging between 500–7.8 µg/mL. After 24 h of incubation, the culture medium was removed, washed using PBS, and incubated (4 h; 37 °C; 5% CO_2_) with 20 µl MTT solution (5 mg/mL). Then, the MTT reaction was stopped by 10% Sodium Dodecyl Sulfate in 0.1 N HCL. Following overnight incubation in a dark environment with a plate covered with aluminum foil, the absorbance was measured using a microplate reader at a wavelength of 595 nm after 10 min of shaking. Three replicates were prepared for each of the three experimental sessions. The following formula calculated the cell viability:

$$\%\;\mathrm{Viable}\;\mathrm{cells}\;=\;\left(\left(\mathrm{treatment}\;\mathrm{group}-\mathrm{medium}\;\mathrm{group}\right)\;/\;\left(\mathrm{Control}\;\mathrm{group}-\mathrm{medium}\;\mathrm{group}\right)\right)\ast100$$

### Statistical analysis

All values were reported as the standard error of the mean (SEM) for all studies, which were conducted in a minimum of two independent experiments with three biological replicates. The Kruskal–Wallis and post hoc Dunn's multiple comparison tests were used to determine difference between mean of the control and treated samples (antibiofilm and antiphospholipase tests). The statistical analyses were performed using GraphPad Prism8 software (GraphPad Software, Inc., La Jolla, CA, USA), and a *p*-value ≤ 0.05 was considered statistically significant.

## Results

### The yields of plants extracts and fractions

The extraction yield reflects the efficiency of the solvent in extracting components from the original matter, a plant powder. Table 1 shows the methanol extract of *A. mangium* leaves with the highest yield at 18.35% and the ethanol extract of *M. pudica* roots with the lowest yield at 7.10%. Furthermore, the ethanol extract of *M. pudica* aerial parts was the most active extract against *C. albicans* biofilm. The ethanol extract (5 g) of *M. pudica* aerial parts was subjected to fractionation by a liquid–liquid partition. We obtained 4.40 g of n-hexane phase (88%), 0.16 g of chloroform phase (3.2%), 0.41 g of ethyl acetate phase (8.2%), 1.39 g of n-butanol phase (27.8%), and 1.26 g of ddH_2_O phase (25.2%). Among these fractions, the lowest yield was observed with the chloroform fraction, whereas the highest was associated with the n-hexane fraction.

**Table 1:** Percentage yields of plant extracts

Samples	Part of plants	Solvents	Powder weight (g)	Extracts weight (g)	Yields (%, w/w)
*Ageratina riparia*	Leaf	Ethanol	100	10.02	10.02
*Mimosa pudica*	Aerial parts	Ethanol	100	7.36	7.36
*Mimosa pudica*	Root	Ethanol	100	7.10	7.10
*Lantana camara*	Leaf	Methanol	100	13.79	13.79
*Mikania micrantha*	Leaf	Methanol	100	15.85	15.85
*Acacia mangium*	Leaf	Methanol	100	18.35	18.35

### Effects of studied invasive plant extracts against *C. albicans* planktonic cells

Table 2 shows the antifungal activity of the studied plant extracts against *C. albicans* ATCC 10231 and the clinical isolates (CI) SPTM and CVX. The ethanol extract of *M. pudica* roots displayed the highest activity against the *C. albicans* ATCC 10231 (MIC_50_ of 7.81 µg/mL) and clinical isolates (MIC_50_ of 15.62—31.25 µg/mL). The ethanol extract of *M. pudica* aerial parts and methanol extract of *A. mangium* leaves showed intermediate activity with MIC_50_ between 15.62 and 62.5 µg/mL. There was no activity of other extracts observed in the present study.

**Table 2:** Susceptibility of *C. albicans* to the studied invasive plant extracts and to fluconazole (MIC and MFC in µg/mL)

Extracts	Pathogens					
	***C. albicans* ATCC 10231**		***C. albicans* CI-SPTM**		***C. albicans* CI-CVX**	
	**MIC**_**50**_	**MFC**	**MIC**_**50**_	**MFC**	**MIC**_**50**_	**MFC**
*A. mangium* leaves	62.5	> 1000	31.25	> 1000	125	> 1000
*A. riparia* leaves	> 1000	NT	NA	NT	> 1000	NT
*M. pudica* aerial parts	15.62	250	62.5	250	62.5	1000
*M. pudica* roots	7.81	125	15.62	125	31.25	250
*L. camara* leaves	> 1000	NT	NA	NT	> 1000	NT
*M. micrantha* leaves	> 1000	NT	NA	NT	> 1000	NT
Fluconazol	0.78	100	1.56	> 200	3.12	> 200
*NA *No Activity, *NT *Not Tested						

### Effects of the studied invasive plant extracts against *C. albicans* biofilms

**Fig. 1:**
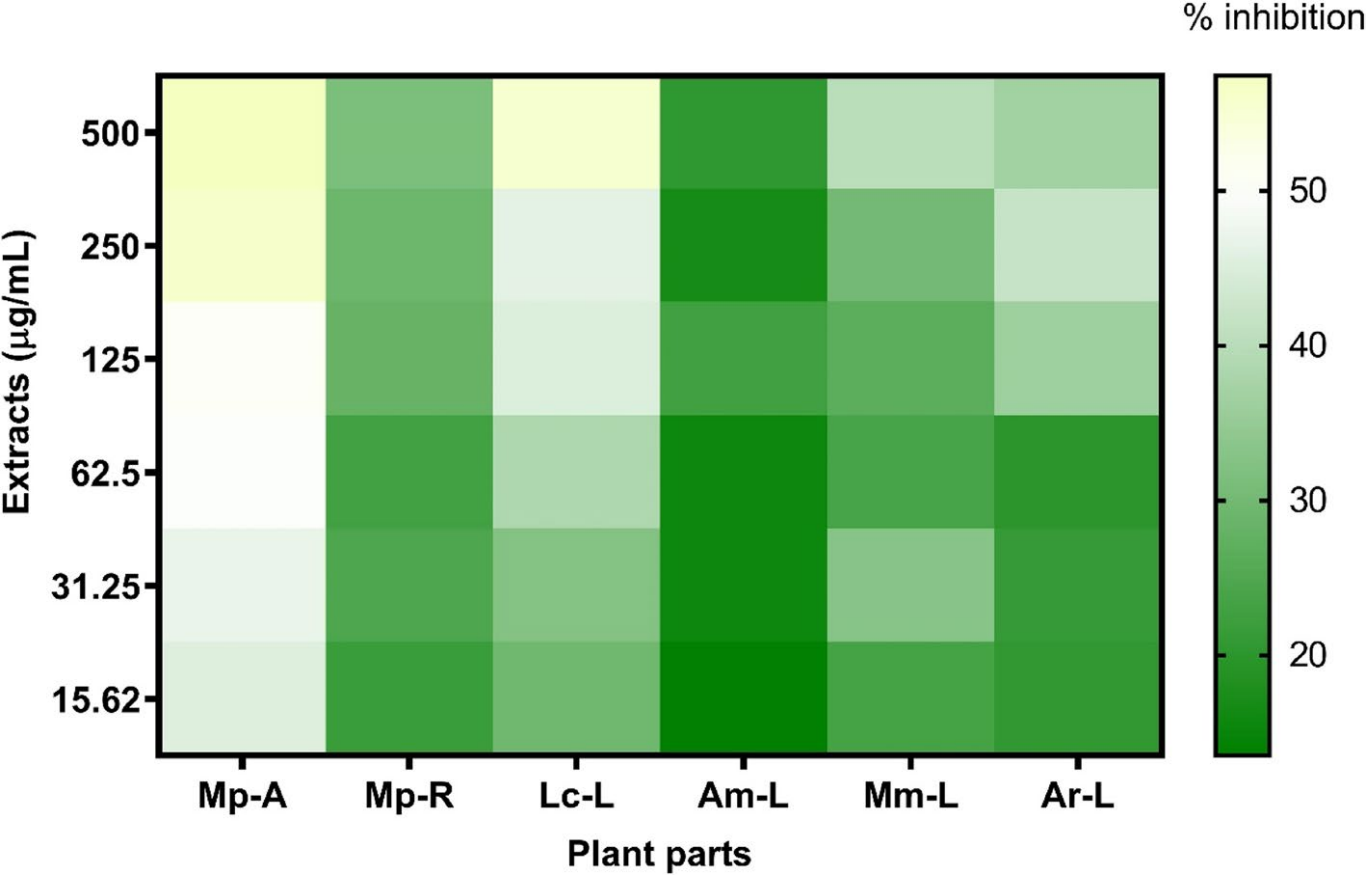
Heat map of the inhibition percentages of the studied extracts against 24 h old biofilm of *C. albicans* ATCC 10231. Mp-A (*M. pudica* aerial parts); Mp-R (*M. pudica* roots); Lc-L (*L. camara* leaves); Am-L (*A. mangium* leaves); Mm-L (*M. micrantha* leaves); Ar-L (*A. riparia* leaves)

All the studied extracts were tested against 24 h old C. *albicans* biofilm. The ethanol extract of *M. pudica* aerial parts was the most active, with 51.11% inhibition at 125 μg mL^−1^ (*p* < 0.05) (Fig. 1). The inhibition of more than 50% was additionally demonstrated by the extract of *L. camara* leaves, however, this was only the case at the highest concentrations (≥ 250 µg/mL). The spectrum activity of a promising extract of *M. pudica* aerial parts was also evaluated against two clinical isolates, CI-SPTM and CI-CVX, and revealed inhibition activity of 53.83% at 125 μg mL^−1^ (p ≤ 0.05) and 50.81% at 250 μg mL^−1^, respectively.

(see Additional file 1).

### Effects of fractions of *M. pudica* aerial parts against 24 h old biofilm of *C. albicans*

**Fig. 2:**
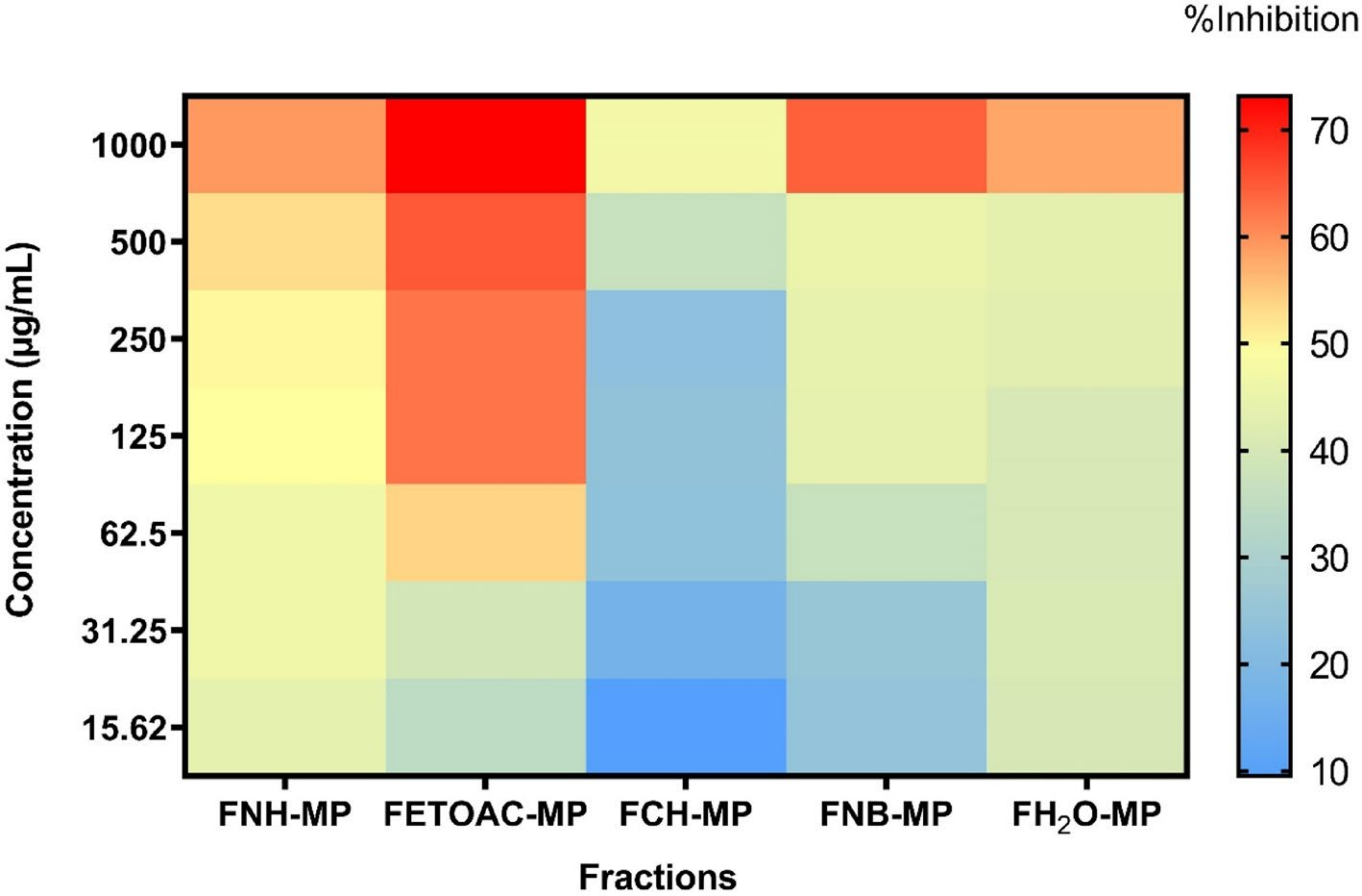
Heat map of the inhibition percentage of all fractions of *M. pudica* aerial parts against 24 h old biofilm of *C. albicans* ATCC 10231. FNH-MP (n-hexane fraction of *M. pudica*); FETOAC-MP (ethyl acetate fraction of *M. pudica*); FCH-MP (chloroform fraction of *M. pudica*); FNB-MP (n-butanol fraction of *M. pudica*); FH_2_O-MP (ddH_2_O fraction of *M. pudica*)

The five obtained fractions from the aerial parts of *M. pudica* were then evaluated against 24 h old biofilm of *C. albicans* ATCC 10231. The results showed that the ethyl acetate fraction of *M. pudica* had the highest activity and inhibited the preformed *C. albicans* biofilms as much as 53.89% at 62.5 μg/mL (*p* ≤ 0.05) (Fig. 2). At the highest concentration (1000 μg/mL), the ethyl acetate fraction inhibited biofilms by over 70%. The hexane fraction inhibited the biofilm cells at a higher concentration than ethyl acetate fraction, by 50.57% at 250 μg/mL. Whereas n-butanol and aqueous fractions showed inhibition of 64.33% and 57.94%, respectively, at the highest concentration (1000 μg/mL), and this activity decreased along with a decrease in the concentration of fractions.

**Fig. 3:**
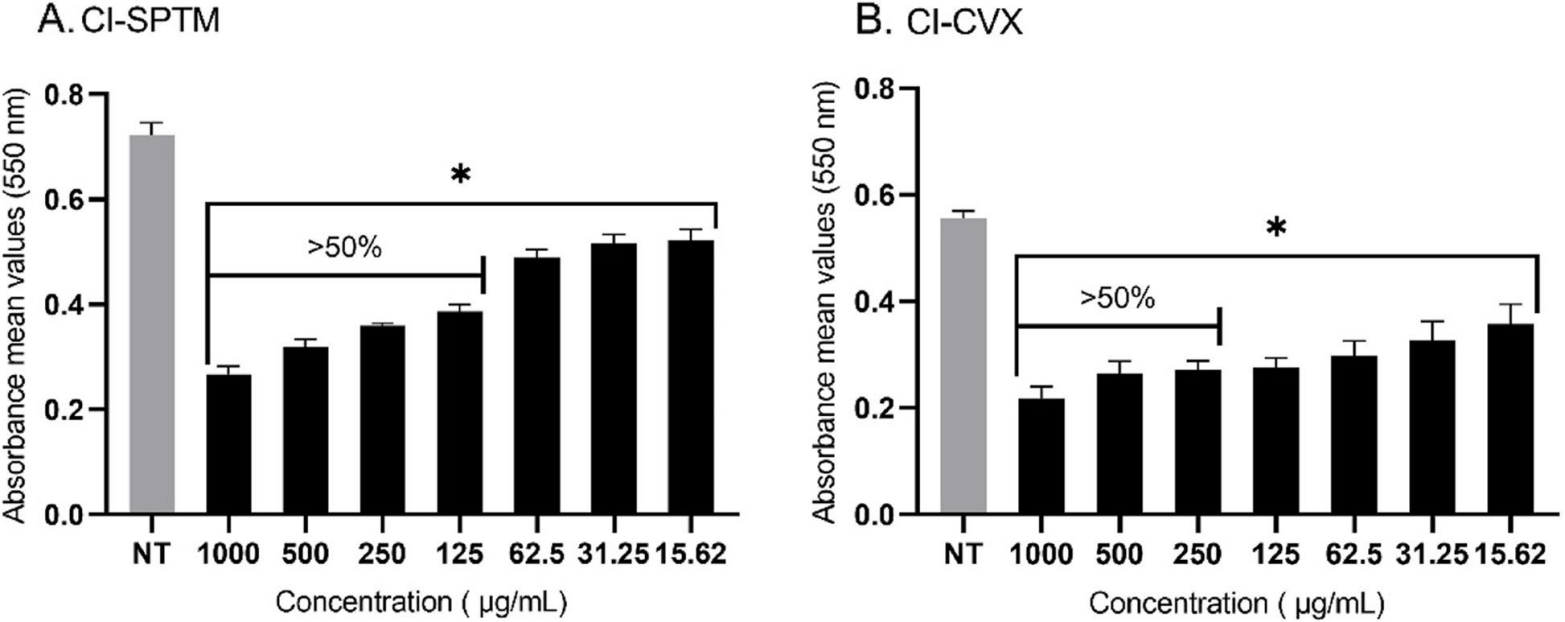
Metabolic activity (MTT assay) of ethyl acetate fraction of *M. pudica* aerial parts against 24 h old clinical isolates (CI-SPTM and C-CVX) biofilms. Asterisks denote statistically significant differences of treated biofilm versus non-treated (NT) biofilm. *p ≤ 0.05 was calculated by Kruskal–Wallis test, followed by Dunn’s multiple comparisons test

The antibiofilm activity of ethyl acetate fraction of *M. pudica* aerial parts was also evaluated against mature biofilm of two clinical isolates: CI-SPTM and CI-CVX. The percentage inhibition on both clinical isolates was approximately 50% at different concentrations (125 vs 250 µg/mL), with CI-SPTM being the most susceptible isolate when treated with ethyl acetate fraction (Fig. 3). The antibiofilm activity is therefore retained in the clinical isolates, but for slightly higher concentrations than in the reference strain.

Alongside antibiofilm activity, we also evaluated the antifungal activity of ethyl acetate fraction of *M. pudica* aerial parts, and we found that the MIC_50_ and MFC were at 31.25 µg/mL and 250 µg/mL against *C. albicans* ATCC 10231, 62.5 µg/mL and 1000 µg/mL against both clinical isolates, respectively.

### Phytochemical composition

#### Ethanol extract of *M. pudica* roots

Demonstrating the most potent antifungal activity, the ethanol extract of *M. pudica* roots was processed for identification and screening of bioactive compounds using LC–MS. Table 3 displays the list of the compounds from the ethanol extract of *M. pudica* roots that have been potentially identified, together with m/z, neutral mass, and retention time. The highest peak was detected at 4.61 retention time and corresponded to the alkaloid compound, 3α-(tigloyloxy) tropane (see Additional file 2). Then, the other ten peaks were chosen for the analysis. However, due to the limitation of the database library (UNIFI software) in the Advanced Characterization Laboratories Serpong, National Research and Innovation Institute (BRIN), some compounds could not be determined (denoted as Candidate mass).

**Table 3:** Mass spectrometric analysis of ethanol extract of *M. pudica* root

Component name	Observed m/z	Neutral mass (Da)	Retention time (min)
Pseudotropidine	142.12	141.11	1.09
Cyclo(Ala-Ala)	143.08	142.07	1.12
Epigallocatechin(4β,8)-gallocatechin	611.14	610.13	1.25
Candidate Mass C_13_H_21_NO_3_	240.16	239.15	4.03
Meteloidine	256.15	255.15	4.41
Candidate Mass C_18_H_27_NO_6_	354.19	353.18	4.51
3α-(Tigloyloxy)tropane	224.16	223.16	4.61
Gallocatechin	329.06	306.07	5.29
3,5,6-Trihydroxy-4',7-dimethoxyflavone	331.08	330.07	5.57
Candidate Mass C_18_H_25_NO_4_	320.19	319.18	6.26
Candidate Mass C_18_H_25_NO_5_	336.18	335.17	6.33

#### Ethyl-acetate fraction of *M. pudica* aerial parts

The most potent antibiofilm activity of ethyl-acetate fraction of *M. pudica* aerial parts was analyzed for its chemical contents using HRMS. Twenty-three and fifty-three compounds were found using HRMS in the negative (Table 4) and positive modes, respectively (Table 5). Because catechin and adenine exhibited the highest peak areas in the negative and positive ionization modes, they were chosen as reference peak areas for calculating relative abundance percentages (RA) [41]. The following prominent compounds in the negative mode were quercetin-3β-D-glucoside (82.17%), (1ξ)-1,5-anhydro-1-[2-(3,4-dihydroxyphenyl)-5,7-dihydroxy-4-oxo-4H-chromen-8-yl]-D-galactitol (52.48%), luteolin (44.24%), and quercetin (24.27%). While in the positive mode were avicularin (47.45%), (1ξ)-1,5-anhydro-1-[2-(3,4-dihydroxyphenyl)-5,7-dihydroxy-4-oxo-4H-chromen-8-yl]-D-galactitol (46.24%), and kaempferol (44.50%).

**Table 4:** Mass spectrometric analysis of ethyl-acetate fraction of *M. pudica* aerial parts using HRMS in negative mode

No	**Name**	**Formula**	**Calculated Molecular Weight (MW)**	**RT [min]**	**Peak Area (Max.)**	**% RA**	**mzCloud Best Match**
1	Chlorogenic acid	C_16_ H_18_ O_9_	354.095	4.284	10,131,770.68	1.65	99.5
2	Catechin	C_15_ H_14_ O_6_	290.079	4.359	613,553,783.2*	100.00	98
3	(1ξ)-1,5-Anhydro-1-[2-(3,4-dihydroxyphenyl)-5,7-dihydroxy-4-oxo-4H-chromen-8-yl]-D-galactitol	C_21_ H_20_ O_11_	448.100	4.545	321,967,998.3	52.48	98
4	(1S,3R,4R,5R)-1,3,4-trihydroxy-5-{[(2E)-3-(4-hydroxy-3-methoxyphenyl)prop-2-enoyl]oxy}cyclohexane-1-carboxylic acid	C_17_ H_20_ O_9_	368.110	5.22	8,509,566.354	1.39	97.6
5	Quercetin-3β-D-glucoside	C_21_ H_20_ O_12_	464.095	5.373	504,131,364.8	82.17	98.4
6	Rutin	C_27_ H_30_ O_16_	610.153	5.467	32,821,476.28	5.35	98.8
7	Syringic acid	C_9_ H_10_ O_5_	198.052	5.673	28,560,131.71	4.65	84.3
8	4,5-Dicaffeoylquinic acid	C_25_ H_24_ O_12_	516.126	6.347	12,801,957.73	2.09	93.5
9	Apigetrin	C_21_ H_20_ O_10_	432.105	6.439	17,355,630.28	2.83	85.4
10	Genistein	C_15_ H_10_ O_5_	270.053	6.517	3,237,563.774	0.53	89
11	4-(3,4-dihydroxyphenyl)-7-hydroxy-5-{[(2S,3R,4S,5S,6R)-3,4,5-trihydroxy-6-(hydroxymethyl) oxan-2-yl]oxy}-2H-chromen-2-one	C_21_ H_20_ O_11_	448.100	6.571	9,208,855.303	1.50	98.4
12	Juglalin	C_20_ H_18_ O_10_	418.090	6.589	27,689,699.26	4.51	93.1
13	NP-015559	C_17_ H_14_ O_7_	330.074	6.869	59,518,391.27	9.70	93.9
14	2,4,6-Trihydroxy-2-(4-hydroxybenzyl)-1-benzofuran-3(2H)-one	C_15_ H_12_ O_6_	288.063	6.971	7,881,929.5	1.28	92.4
15	Luteolin	C_15_ H_10_ O_6_	286.047	7.355	271,424,429.2	44.24	92.4
16	3-tert-Butyladipic acid	C_10_ H_18_ O_4_	202.120	7.609	6,456,519.47	1.05	80.4
17	Quercetin	C_15_ H_10_ O_7_	302.042	7.654	148,931,203.7	24.27	97.6
18	Eriodictyol	C_15_ H_12_ O_6_	288.063	7.746	10,578,247.87	1.72	73.1
19	3-Methoxy-5,7,3',4'-tetrahydroxy-flavone	C_16_ H_12_ O_7_	316.058	8.023	47,968,532.18	7.82	99.1
20	NP-019001	C_18_ H_12_ O_7_	340.058	8.286	39,751,166.39	6.48	76.1
21	Corchorifatty acid F	C_18_ H_32_ O_5_	328.225	8.355	80,920,096.63	13.19	97.1
22	(15Z)-9,12,13-Trihydroxy-15-octadecenoic acid	C_18_ H_34_ O_5_	330.240	8.775	30,194,667.16	4.92	85.2
23	2,2'-Methylenebis(4-methyl-6-tert-butylphenol)	C_23_ H_32_ O_2_	340.240	16.552	22,234,098.55	3.62	96.3

Relative percentage abundance (% RA) was measured by the ratio RA of the given peak area to RA of the *reference peak

**Table 5:** Mass spectrometric analysis of the ethyl-acetate fraction of *M. pudica* aerial parts using high-resolution mass spectrometry in positive mode

No	**Name**	**Formula**	**Calc. MW**	**RT [min]**	**Area (Max.)**	**% RA**	**mzCloud Best Match**
1	Choline	C5 H13 N O	103.0999	0.963	38,986,744.01	5.70	95.7
2	D-( +)-Proline	C5 H9 N O2	115.0634	1.016	19,894,456.59	2.91	92.2
3	Adenine	C_5_ H_5_ N_5_	135.0543	1.028	684,067,887*	100.00	99.4
4	NP-019811	C_6_ H_7_ N O_2_	125.0477	1.037	237,023,365	34.65	95.4
6	Pyrrole-2-carboxylic acid	C5 H5 N O2	111.0322	1.047	54,258,960.28	7.93	95
7	Tropine	C8 H15 N O	141.1153	1.056	60,269,180.57	8.81	99.5
8	3-Hydroxypyridine	C5 H5 N O	95.03736	1.064	178,293,700.5	26.06	100
9	Pyridoxine	C8 H11 N O3	169.0737	1.112	48,875,824.48	7.14	99.4
10	NP-000358	C15 H14 O7	306.0737	1.139	24,522,474.57	3.58	99.5
11	L-Isoleucine	C6 H13 N O2	131.0946	1.562	9,498,791.612	1.39	90.4
12	Nicotinamide	C6 H6 N2 O	122.048	1.616	23,108,176.84	3.38	91
13	Nicotinic acid	C6 H5 N O2	123.032	1.618	32,845,375.37	4.80	95
14	N, N-Dimethylaniline	C8 H11 N	121.0892	3.791	31,350,071.07	4.58	86.7
15	(1ξ)-1,5-Anhydro-1-[2-(3,4-dihydroxyphenyl)-5,7-dihydroxy-4-oxo-4H-chromen-8-yl]-D-galactitol	C21 H20 O11	448.0997	4.601	316,295,296.2	46.24	96.7
16	5-Methoxysalicylic acid	C8 H8 O4	168.0422	4.732	12,290,077.79	1.80	77.2
17	Scopoletin	C10 H8 O4	192.0422	5.049	20,362,873.88	2.98	92.5
18	Esculetin	C9 H6 O4	178.0265	5.105	14,215,495.18	2.08	89.9
19	Cynaroside	C21 H20 O11	448.0997	5.288	122,295,357.9	17.88	99
20	(1S)-1,5-Anhydro-2-O-(6-deoxy-α-L-mannopyranosyl)-1-[5,7-dihydroxy-2-(4-hydroxyphenyl)-4-oxo-4H-chromen-6-yl]-D-glucitol	C27 H30 O14	578.1632	5.448	22,312,282.07	3.26	93.5
21	NP-021018	C12 H18 O4	226.1203	5.455	40,180,312.67	5.87	73.9
22	Toliprolol	C13 H21 N O2	223.157	5.471	44,615,504.37	6.52	71.5
23	Rutin	C27 H30 O16	610.1529	5.481	12,773,553.55	1.87	98.8
24	Jasmonic acid	C12 H18 O3	210.1254	5.587	5,903,842.209	0.86	73.1
25	1,5-Anhydro-1-[5,7-dihydroxy-3-(4-hydroxyphenyl)-4-oxo-4H-chromen-8-yl]hexitol	C21 H20 O10	432.1053	5.614	170,435,176.3	24.91	72.9
26	Quercetin-3β-D-glucoside	C21 H20 O12	464.095	5.737	163,476,631.8	23.90	99.6
27	Quercetin	C15 H10 O7	302.0422	5.751	165,098,283.2	24.13	98.5
28	NP-018720	C27 H28 O16	608.1373	5.986	6,274,504.294	0.92	97
29	Avicularin	C_20_ H_18_ O_11_	434.0843	6.131	324,561,610.4	47.45	99.7
30	Aflatoxin G1	C_17_ H_12_ O_7_	328.0578	6.187	200,501,817.2	29.31	86
31	Diosmetin	C16 H12 O6	300.063	6.206	71,678,113.21	10.48	87
32	4-Coumaric acid	C9 H8 O3	164.0473	6.213	23,341,562.64	3.41	91.5
33	NP-015559	C17 H14 O7	330.0734	6.384	61,595,289.75	9.00	90
34	Vitexin	C21 H20 O10	432.1053	6.443	36,425,664.32	5.32	76.5
35	Galangin	C15 H10 O5	270.0525	6.531	9,846,563.576	1.44	98.7
36	5,7-Dihydroxy-2-(4-hydroxyphenyl)-4-oxo-4H-chromen-3-yl β-L-xylofuranoside	C20 H18 O10	418.0895	6.602	28,743,146.4	4.20	94.5
37	Isokaempferide	C16 H12 O6	300.063	6.784	4,774,062.327	0.70	87.4
38	5-O-Methylgenistein	C16 H12 O5	284.0682	6.838	11,697,238.21	1.71	98.7
39	NP-003294	C18 H16 O7	344.0893	6.859	6,715,576.313	0.98	83.1
40	NP-000465	C17 H14 O6	314.0787	6.941	30,654,410.59	4.48	90.7
41	(-)-Caryophyllene oxide	C15 H24 O	220.1826	7.153	25,344,135.34	3.70	83.2
42	Chrysin	C15 H10 O4	254.0577	7.241	11,993,651.12	1.75	98.5
43	Kaempferol	C_15_ H_10_ O_6_	286.0474	7.341	304,396,847.3	44.50	99.4
44	NP-021018	C12 H18 O4	226.1203	7.36	13,126,756.27	1.92	76.9
45	Aflatoxin G2	C17 H14 O7	330.0734	7.365	5,843,945.045	0.85	81.8
46	N-(2,4-Dimethylphenyl) formamide	C9 H11 N O	149.084	7.814	44,191,081.65	6.46	93.3
47	3-Methoxy-5,7,3',4'-tetrahydroxy-flavone	C16 H12 O7	316.0581	8.042	32,003,335.9	4.68	99
48	9S,13R-12-Oxophytodienoic acid	C18 H28 O3	292.2035	8.326	18,388,834.54	2.69	85.9
49	Apigenin	C15 H10 O5	270.0525	8.45	53,092,646.63	7.76	99.8
50	α-Pyrrolidinopropiophenone	C13 H17 N O	203.1311	13.213	9,792,651.46	1.43	92.7
51	Stearamide	C18 H37 N O	283.2875	16.424	15,774,788.86	2.31	97.4
52	Hexadecanamide	C16 H33 N O	255.2561	14.821	8,241,911.601	1.20	86.5
53	Oleamide	C18 H35 N O	281.2717	15.321	8,537,558.88	1.25	96

### Effects of the ethanol extract of *M. pudica* aerial parts against phospholipase activity

**Fig. 4:**
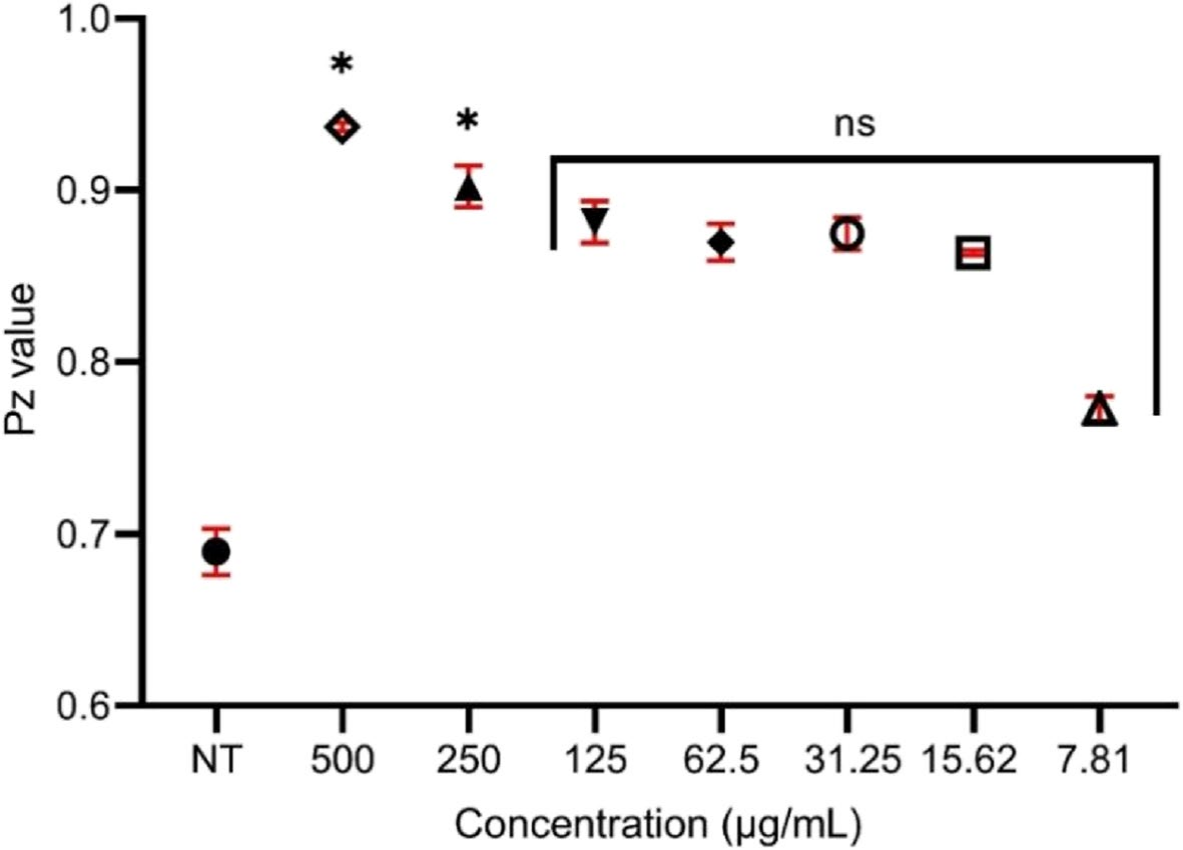
The effect of ethanol extract of *M. pudica* aerial parts on the production of phospholipases secreted by *C. albicans* ATCC 10231. *p ≤ 0.05 and ns (not significant) were calculated by the Kruskal–Wallis test, followed by Dunn’s multiple comparison test

When *C. albicans* cells (control group) were cultured on the surface of egg-yolk emulsion agar (phospholipase induction), the average value of the phospholipases was 0.69 ± 0.013, demonstrating that the control group released many phospholipases. The mean extracellular phospholipases activity (Pz index) in the cells treated with the ethanol extracts of *M. pudica* aerial parts at 500 µg/mL and 250 µg/mL, were 0.94 ± 0.002 and 0.90 ± 0.012, respectively (Fig. 4). The extract decreased phospholipase activity significantly at these concentrations. At lower concentrations (125 µg/mL to 7.81 µg/mL), the reduction of phospholipase activity was statistically insignificant (*p* > 0.05).

### Scanning electron microscopy observations of the effects of the ethyl-acetate fraction of *M. pudica* aerial parts on *C. albicans* biofilm

**Fig. 5:**
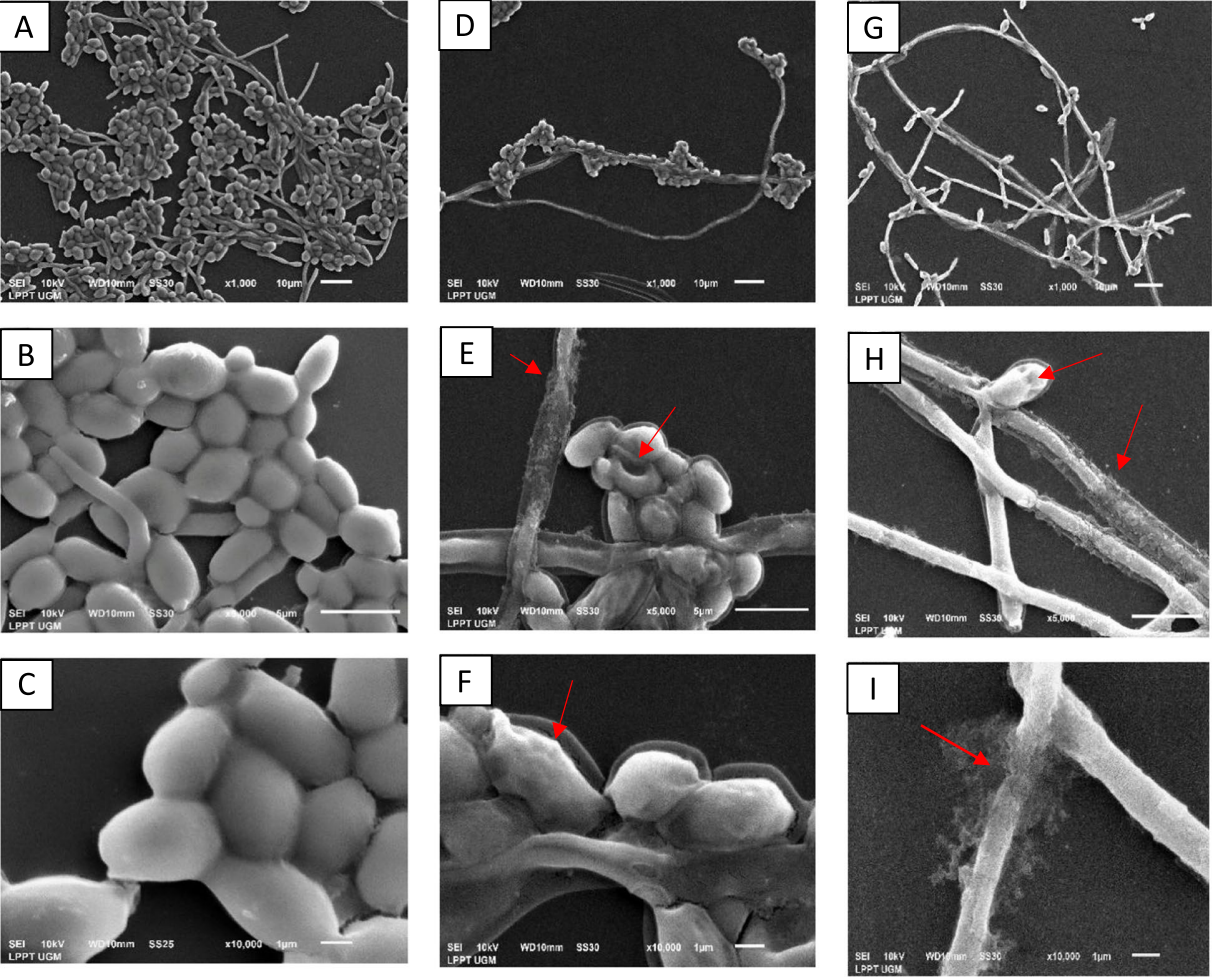
Scanning electron microscopy of *C. albicans* ATCC 10231 24 h old biofilm with or without treatment with ethyl-acetate fractions of *M. pudica* aerial parts. The control group (**A**-**B**-**C**) was treated with 62.5 µg/mL (**D**-**E**–**F**) and 125 µg/mL (**G**-**H**-**I**). The arrows pointed to some surface morphological changes

This current work employed scanning electron microscopy (SEM) to investigate the effect of the ethyl acetate fraction of *M. pudica* aerial parts on the surface morphology of 24 h old *C. albicans* biofilm. The control cells (without fraction) showed a smooth, regular colony cell shape and a distinct bud morphology, as seen in Fig. 5 from A to C. In contrast to the untreated cells, those treated by the fraction showed an irregular cell shape, rough surface collapses, and disrupted hyphae (Fig. 5D–I).

### Cytotoxic activity of ethyl-acetate fraction of *M. pudica* aerial parts

**Fig. 6:**
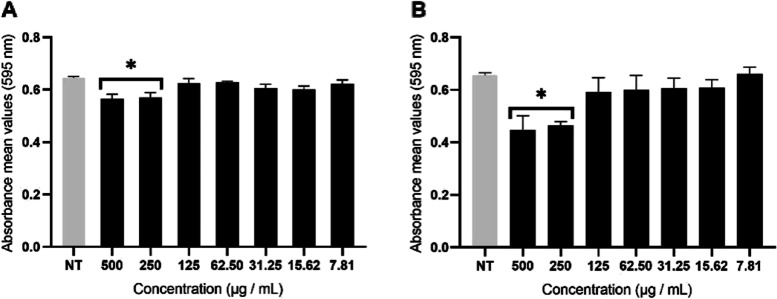
Effect of ethyl acetate fraction of *M. pudica* aerial parts on the metabolic activity (MTT) of Vero (**A**) and HeLa (**B**) cells after 24 h treatment. Error bars indicate the standard error of the mean of three independent experiments performed in triplicate. *p < 0.05 calculated by Kruskal Wallis, followed by Dunn's multiple comparison test

It was evaluated that the ethyl acetate fraction of *M. pudica* aerial parts was not cytotoxic when tested at the concentration corresponding to its biofilm inhibitory concentration (Fig. 6). Result indicates that at the biofilm inhibitory concentration (50%), 96.77% of Vero cells and 89.91% of HeLa cells were viable. While at the greatest concentration, 500 μg/mL, the viability of Vero and HeLa cells was 61.86% and 85.66%, respectively.

## Discussion

The ability of *C. albicans* to grow as a community of adherent cells encapsulated by extracellular matrix puts well-known antifungal drugs at risk of resistance, growing interest of drug discovery by utilizing plants that have existed for millions of years. Plants synthesize secondary metabolites or natural products that are generally divided into three classes, including phenolics, terpenoids, and alkaloids. Numerous studies employed natural products from native plants to combat biofim-induced fungal resistance [42–45]. However, exploring natural products from invasive plants as antibiofilm candidate drugs is still limited.

In this study, we used five invasive plants that were extracted by a maceration method using methanol or ethanol solvents. Furthermore, the most active extract was separated by the LLF method, and solvents were selected based on their degree of polarity. According to the results of the extraction method, methanol provided a greater yield contribution than ethanol. Even though we did not extract every studied plant with a variety of polar solvents, it is possible to hypothesize that increasing the polarity of the solvents might enhance the extraction yields. It means that in this study, methanol was more efficient at extracting phytochemicals of plants than ethanol. This is consistent with what was reported in previous research, which demonstrated that the yield of some plants, such as *A. mangium* leaf extract, *M. pudica* aerial parts, and *Vernonia auriculifera* Hiern leaves, was the greatest when it was extracted in a polar solvent [46–48]. However, the result of the fractionation yield of *M. pudica* in this study revealed that even though n-hexane is the lowest polarity solvent, it was associated with the highest yield. This finding indicates that most of the substances in *M. pudica* extract were non-polar substances. It needs to be emphasized, the differences in the extraction/fractionation yield are influenced by several factors, including not only by solvent polarity or typet, but also by extraction or fractionation method, the size of material, extraction time, and temperature [47, 49].

According to our results, the ethanol extracts of *M. pudica* (aerial parts and roots) exhibited great power of antifungal activity against *C. albicans* ATCC 10231 with MIC_50_ of 15.62 µg/mL and 7.81 µg/mL, respectively. Even though the MIC_50_ was 10–20 fold higher than that of fluconazole, both extracts exhibited comparable fungistatic rather than fungicidal properties to fluconazole. In previous studies, *M. pudica* extracts showed antifungal activity with various activity levels. It was reported that methanol extract from the leaves of invasive *M. pudica* growing in India showed an antifungal effect against *C. albicans* with MIC ranging between 0.394 and 0.398 mg/mL [50]. Two other studies evaluated the *M. pudica* antifungal activity by an agar disk diffusion method: the first one reported that ethanol extract of *M. pudica* leaves was effective against *C. albicans* at 30 mg/mL with a zone of inhibition of 17 mm [51] and the second study reported that *M. pudica* fractions and its diterpenoids, named 19-*O*-transferuloyl-labd-8(17)-en-15,19-diol and 19-*O*-[(E)-3’,4’-dimethoxy cinnamoyl]-labd-8(17)-en15,19-diol, inhibited *C. albicans* with an inhibition zone ranging from 9–15 mm [52]. Overall, the MICs of *M. pudica* extracts in our study against *C. albicans* were lower than those reported in the literature. It is noteworthy that it is difficult to compare our results with the reported literature because of the variations in the utilization of solvent, extraction/fractionation process, and antifungal method. In addition, it is conceivable that the chemical content of invasive *M. pudica* plants cultivated in Indonesia differs from those grown in other countries.

The antifungal activity of the root extract of *M. pudica* was mainly influenced by the bioactive compounds that either function independently, in synergy, or antagonistically with the other compounds. The LC–MS/MS analyses suggested that alkaloids are secondary plant metabolites that might be responsible for antifungal activity. However, we cannot disallow the possibility that existing flavonoids in the extract might also have this effect. To our best knowledge, no literature has yet reported these compounds present in the extract from the root of *M. pudica*. However, some compounds of tropane alkaloid were found in *Datura stramonium*, and particularly, 3*α*-tigloyloxytropane has been found in the variant *D. Stramonium* grown in Egypt [53]. But, no literature reported the antifungal activity of a 3*α*-tigloyloxytropane against *C. albicans*. The compounds of polyphenols (epigallocatechin(4β,8)-gallocatechin, and gallocatechin) and flavonoid (3,5,6-trihydroxy-4',7-dimethoxyflavone) which present in our study*,* might be contributing to the antifungal activity against *C. albicans*. The anti-*Candida* properties of polyphenol compounds have been reported in the literature: Evensen and Braun, 2009 demonstrated that phenolic compounds in green tea extracts reduced by 43% the growth of *C. albicans* when used at 5 mg/mL [54]. Other studies reported that proanthocyanidins (oligomeric flavonoids composed by derivatives of catechin and epicatechin and their gallic acid esters) polymer-rich fractions from the stem bark of *Stryphnodendron adstringens* revealed antifungal activity against *C. albicans* with MIC values of 15.6 μg/mL [55]. Finally, the mixture of epigallocatechin, gallocatechin, and epigallocatechin-(4β → 8)-gallocatechin in the subfractions from the stem bark of *Stryphnodendron obovatum Benth* showed antifungal activity against *C. albicans* and *C. parapsilosis* with MIC ranging from 31.5 µg/mL to 125 µg/mL [56].

The ethyl acetate fraction obtained from the aerial parts of *M. pudica* had good activity against planktonic cells of *C. albicans* ATCC 10231. The HR-MS analyses of this fraction revealed the presence of several kinds of compounds and, in particular, flavonoids such as avicularin, quercetin, luteolin, rutin, kaempferol, catechin, quercetin-3β-D-glucoside, and 1ξ-1,5-anhydro-1-[2-(3,4-dihydroxyphenyl)-5,7-dihydroxy-4-oxo-4H-chromen-8-yl]-D-galactitol. It is known that flavonoids have an intermediate polarity, making them extractable with ethyl-acetate [57]. They are also linked to multiple antifungal pathways, including disturbance of the plasma membrane, stimulation of mitochondrial malfunction, suppression of cell structural work, cell division, RNA and protein synthesis, and efflux mediated pumping systems [58, 59].

Several studies demonstrated the presence of flavonoids in invasive *M. pudica* plant: Sapkota et al*.* reported that an ethyl-acetate fraction of *M. pudica* growing in Nepal contained quercetin, catechin, and avicularin [60]; Yusof et al.determined orientin, kaempferol 7-rutinoside, and kaempferol 3-glucoside-7-rhamnoside in *M. pudica* aerial parts [61]; and Lobstein et al*.* isolated myricetin and two C-glycosylflavones, 4″-hydroxymaysin and cassiaoccidentalin B, from *M. pudica* aerial parts [62].

Literature reported that avicularin, kaempferol, luteolin, and quercetin inhibited the growth of planktonic *Candida* species [63–66]. Furthermore, it has been established that quercetin inhibited fatty acid synthase, an enzyme essential for endogenous fatty acid production in the fungal membrane, as part of its antifungal action [42]. In addition, quercetin induced apoptosis of *C. albicans* by increasing intracellular Mg2 + , mitochondrial Ca2 + , and mitochondrial dysfunction, which triggers the decline in mitochondrial redox levels and disruption in the mitochondrial antioxidant system [67].

Concerning the antibiofilm activity, the ethyl-acetate fraction of *M. pudica* aerial parts inhibited the metabolic activity of 24 h old biofilms of *C. albicans*. To the best of our knowledge, this is the first study describing the antibiofilm activity of *M. pudica*. The ability of *M. pudica* to inhibit biofilm formation was previously described only against a single-species bacterial biofilm, *Streptococcus mutans* biofilm [68]. Related to the effects of flavonoids against *Candida* biofilms, it has been reported that kaempferol inhibited *C. albicans* biofilm by reducing the hyphal formation and hydrophobicity of the fungal cell surface [43]. Another study showed that kaempferol and quercetin diminished the biomass of *C. orthopsilosis* and *C. metapsilosis* and the metabolic activity and biomass of developing biofilms of the *C. parapsilosis* complex [65]. Also, cathechin inhibited the biofilm formation of *C. albicans* involving proteasomal enzyme activity leading to metabolic instability and membrane cell disruption [54]. Concerning luteolin, this flavonoid doesn't seem like a good antibiofilm candidate, as a high concentration (625–5000 µg/mL) was required to prevent the formation of *C. albicans* biofilms [66]. Based on the literature, it was speculated that the activity of studied *M. pudica* against 24 h old *C. albicans* biofilm was due to the presence of flavonoids. However, the possible implications of other existing components in the present study are still required to determine the activity. For example, the presence of terpenes or terpenoids in our studied plant might play a role in the antibiofilm activity. Several studies have reported that terpenes showed antifungal [69] and antibiofilm activity [44, 70]. Indeed, work of Spengler et al., 2022 demonstrated the antibiofilm mechanism via efflux pump inhibitory on some bacteria [44].

Regarding the biofilm structure, SEM observations showed that the ethyl-acetate fraction of *M. pudica* aerial parts (at 62.5 and 125 µg/mL) influenced the surface morphology of *C. albicans* cells, and notably, no cytotoxic effect on the Hela and Vero cells evaluated at these concentrations. This finding demonstrated a qualitative correlation between the biofilm observed by microscopy and metabolic activity. After observing the effects of *M. pudica* on the growth of *C. albicans* cells in planktonic and biofilm modes, the possible action of the extract of *M. pudica* aerial parts in inhibiting the secretion of phospholipase enzyme was evaluated. The production of phospholipase enzyme is a fundamental event in the pathogenesis of *C. albicans* during the adhesion and invasion stages by damaging and penetrating host cell membranes, promoting blastospore hyphal development, etc.. [11, 71]. Our results showed that *M. pudica* aerial parts reduced phospholipase secretion, but it was only significant at high concentrations. To the best of our knowledge, this was the first study to report the effects of ethanol extract *M. pudica* on phospholipase enzyme.

## Conclusions and future directions

The screening of the activity of five invasive plant extracts grown in Indonesia against *C. albicans* biofilms highlighted the interest in the ethyl acetate fraction of *M. pudica* aerial parts. To the best of our knowledge, this is the first study to investigate the effects of *M. pudica* on virulence factors, particularly against *C. albicans* biofilm. Even though ethanol extracts of *M. pudica* aerial parts and roots showed good antifungal activity, since the main objective of this work was to find antibiofilm compounds, we did not deeply investigate the antifungal potential of those extracts. However, this promising activity encourages further study, starting with testing it on other clinical strains and fungal species of *Candida* to clarify its spectrum of antifungal activity. Meanwhile, even though flavonoids in the ethyl acetate fraction of *M. pudica* aerial parts might exert antibiofilm activity, other components cannot be ignored. Thus, some steps can be taken, including 1} chemical characterization should be applied along with the evaluation of antibiofilm activity (bioassay-guided isolation). Therefore, the active pure compound can be determined. Another fast track to determining active pure compound is by 2} using bio-chemometric study, an interdisciplinary research field involving multivariate statistics, mathematical modeling, and computing, and is particularly applied to understanding chemical data, specifically in this term for the antibiofilm activity of bioactive compounds.

## Supplementary Information

**Additional file 1.**

**Additional file 2.**

## Data Availability

The data used to support the findings of this study are available from the corresponding author upon request.
