# Supplementary material for: Screening antibiofilm activity of invasive plants growing at the Slope Merapi Mountain, Central Java, against Candida albicans
Source: BMC Complement Med Ther. 2023 Jul 12;23:232. doi: 10.1186/s12906-023-04044-2 (PMC10339508; doi:10.1186/s12906-023-04044-2)

Metabolic activity (MTT assay) of ethanol extract of *M. pudica* aerial parts against 24 h old clinical isolates SPTM and CVX biofilms. Error bars indicate the standard error of the mean of three independent experiments performed in triplicate. *p < 0.05 calculated by Kruskal Wallis, followed by Dunn's multiple comparison test.

1. **Clinical isolate CVX**
2. **Clinical isolate SPTM**


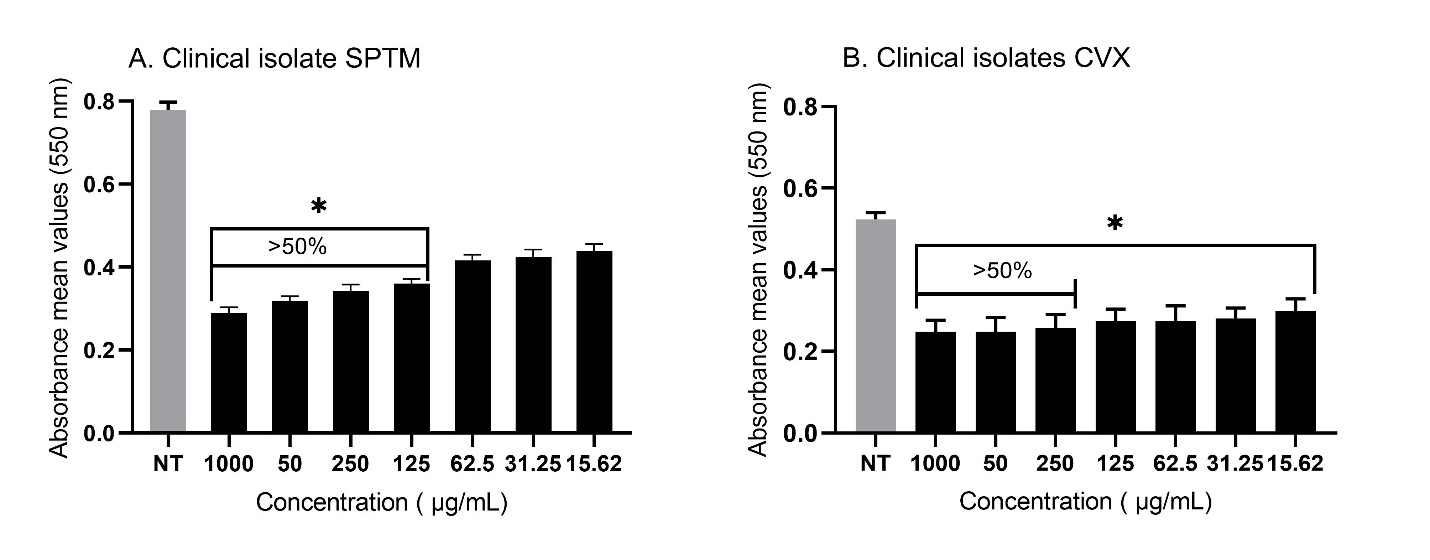

Supplement: Supplementary file 1 — Additional file 1. [file 12906_2023_4044_MOESM1_ESM.docx]
