# Supplementary figures and images for: Screening antibiofilm activity of invasive plants growing at the Slope Merapi Mountain, Central Java, against Candida albicans
Source: BMC Complement Med Ther. 2023 Jul 12;23:232. doi: 10.1186/s12906-023-04044-2 (PMC10339508; doi:10.1186/s12906-023-04044-2)

Additional file 2

LC-ESI-QTOF-MS/MS peak chromatogram of the ethanol extract of *M. pudica* roots.


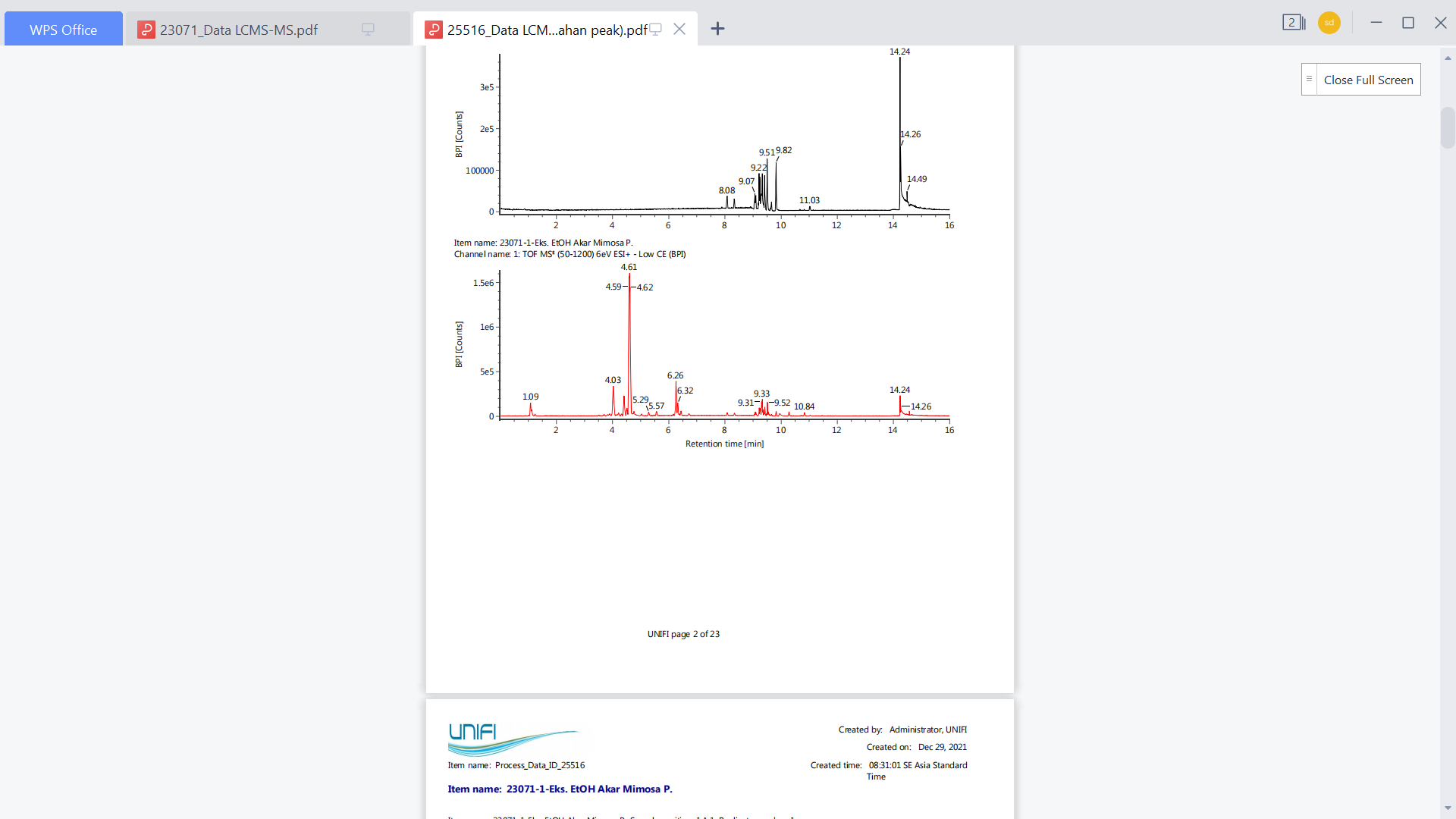

Supplement: Supplementary file 2 — Additional file 2. [file 12906_2023_4044_MOESM2_ESM.docx]
